# Mathematical modeling of endogenous and exogenously administered T cell recirculation in mouse and its application to pharmacokinetic studies of cell therapies

**DOI:** 10.3389/fimmu.2024.1357706

**Published:** 2024-04-17

**Authors:** Antonina Nikitich, Gabriel Helmlinger, Kirill Peskov, Gennady Bocharov

**Affiliations:** ^1^ Research Center of Model-Informed Drug Development, I.M. Sechenov First Moscow State Medical University, Moscow, Russia; ^2^ Marchuk Institute of Numerical Mathematics of the Russian Academy of Sciences (INM RAS), Moscow, Russia; ^3^ Biorchestra US Inc., Cambridge, MA, United States; ^4^ Modeling and Simulation Decisions FZ - LLC, Dubai, United Arab Emirates; ^5^ University of Science and Technology (STU) “Sirius”, Sochi, Russia; ^6^ Institute for Computer Science and Mathematical Modelling, I.M. Sechenov First Moscow State Medical University, Moscow, Russia; ^7^ Moscow Center of Fundamental and Applied Mathematics at INM RAS, Moscow, Russia

**Keywords:** PBPK model, T cell migration, cellular kinetics, adoptive T cell transfer, T cell homeostasis

## Abstract

**Introduction:**

In vivo T cell migration has been of interest to scientists for the past 60 years. T cell kinetics are important in the understanding of the immune response to infectious agents. More recently, adoptive T cell therapies have proven to be a most promising approach to treating a wide range of diseases, including autoimmune and cancer diseases, whereby the characterization of cellular kinetics represents an important step towards the prediction of therapeutic efficacy.

**Methods:**

Here, we developed a physiologically-based pharmacokinetic (PBPK) model that describes endogenous T cell homeostasis and the kinetics of exogenously administered T cells in mouse. Parameter calibration was performed using a nonlinear fixed-effects modeling approach based on published data on T cell kinetics and steady-state levels in different tissues of mice. The Partial Rank Correlation Coefficient (PRCC) method was used to perform a global sensitivity assessment. To estimate the impact of kinetic parameters on exogenously administered T cell dynamics, a local sensitivity analysis was conducted.

**Results:**

We simulated the model to analyze cellular kinetics following various T cell doses and frequencies of CCR7+ T cells in the population of infused lymphocytes. The model predicted the effects of T cell numbers and of population composition of infused T cells on the resultant concentration of T cells in various organs. For example, a higher percentage of CCR7+ T cells among exogenously administered T lymphocytes led to an augmented accumulation of T cells in the spleen. The model predicted a linear dependence of T cell dynamics on the dose of adoptively transferred T cells.

**Discussion:**

The mathematical model of T cell migration presented here can be integrated into a multi-scale model of the immune system and be used in a preclinical setting for predicting the distribution of genetically modified T lymphocytes in various organs, following adoptive T cell therapies.

## 1 Introduction

Processes related to the migration and circulation of lymphocytes are critical for the functioning of the immune system and have been extensively studied over the past 60 years (1–3). Initially, T cells are produced in the thymus. Upon positive and negative selection, they reach the systemic circulation (4). A portion of naïve and effector T cells migrate from blood to tissue parenchyma (5); another portion of T cells migrate to lymph nodes (LNs) via the afferent lymphatic vessels, which collect lymph from non-lymphoid organs, although a majority of T cells enter LNs through high endothelial venules (HEVs) (6). This process is controlled by the expression of specific chemokine receptors on the surface of T cells, and only CCR7^+^ T cells may enter LNs through HEVs (7). In lymphoid tissue, T cells may undergo homeostatic proliferation in the absence of antigen or interact with antigen-presenting cells to get activated, proliferate (in the presence of antigen) and/or undergo apoptosis, and then leave LNs through efferent lymphatic vessels (8). Upon leaving the LNs via lymphatic vessels, all T cells flow to the thoracic duct, which returns them into the systemic circulation (9). The above scheme represents the most basic steps of T cell trafficking in the organism; these are further modulated via numerous and complex molecular and cellular interactions.

T cells are the main effector cells in the adaptive immune response and have been increasingly considered for use as therapeutic agents. Currently, such therapeutic interventions are based on the infusion of autologous or genetically modified T cells, such as tumor-infiltrating lymphocytes (TILs), gene-modified T cells expressing novel T cell receptors (TCR-based therapies), chimeric antigen receptor T cells (CAR-T therapies), CAR-NK therapies, and other treatment modalities. The multiple molecular modifications that occur in the immune engineering and manufacturing of T cell therapies significantly affect T cell migration characteristics, once these cells are infused back *in vivo*, thereby leading to specific T cell pharmacokinetic patterns and subsequent targeted and non-targeted pharmacological responses. A detailed, quantitative understanding of the migration processes of mixed population of exogenously delivered and endogenous T cells is, therefore, of fundamental importance in the development and optimization of translational and clinical strategies for such T cell-based therapies.

A multitude of *in vitro* and *in vivo* studies have been conducted, in the investigation of processes that regulate T lymphocyte migration. One foundational experimental analysis has been reported by Smith and Ford (10). In related studies, rats received radiolabeled thoracic duct lymphocytes intravenously, for an evaluation of radioactivity in various organs. Another series of foundational experiments has been performed by Sprent and Miller, who conducted studies in mice treated with labeled T or B cells (11, 12). Subsequently, the utilization of radiolabeled lymphocytes emerged as the predominant method for the *in vivo* examination of immune cell migration and distribution (13, 14). A number of recent studies have been reported, whereby T cell distribution in different tissues was quantitatively determined through Cr^51^-radiolabeled T cell kinetics (15), following adoptive therapy T cell administration (activated or gene-modified T cells) (16, 17).

Given the high degree of complexity of T cell migration processes - including numerous, intricate and nonlinear dynamic interactions - there have been multiple attempts to describe these mathematically. Mohler and Farooqi (18) and later Ganusov and Auerbach (19) described T cell migration processes based on data from experiments of Smith and Ford (10), using physiologically-based compartmental models. Specifically, Mohler and Farooqi (18) developed a family of related mathematical models. One model was formulated with delay differential equations (DDEs) and four other models made use of ordinary differential equations (ODEs) with variations in their structure, i.e., linear and non-linear models, time-variant and time-invariant models. In their model, the authors proposed a compartmental structure whereby blood plasma was considered as a central compartment, while other organs were secondary in terms of lymphocyte migration. Ganusov and Auerbach used their model to estimate lymphocyte residence time in various organs and proposed hypotheses about the mechanisms of lymphocyte arrest in LNs during activation (19). The limitation of the mathematical models calibrated using the data from the Smith and Ford studies relates to the lack of information on absolute numbers of lymphocytes, since the data and corresponding variables in the models were characterized as “percentages of the injected dose”, while the dose of infused lymphocytes was not explicitly provided. Later, Ganusov and Tomura used the same approach to describe *in vivo* T cell recirculation as a function of exogenously administered T cell kinetics (20). In that model, T cell migration in LNs was described with reference to cell fluorescence data measured in Kaede mice (21). Additional compartments were considered to describe the delay in T cell transition through LNs, and a two-compartment model was finally considered as optimal.

To investigate T cell migration through a specific organ such as the spleen or LNs, a set of experiments was conducted to further develop models of lymphocyte migration (22, 23). Later, Khot et al. (15) and Singh et al. (24) used a multi-compartmental, physiologically-based pharmacokinetic (PBPK) approach to develop mathematical models of T cell recirculation. In such models, each organ was typically divided into extravascular and intravascular spaces. This division allowed one to model the transition of T cells through a given organ, their exit from the blood circulation, and their migration through the lymphatic system. These models described the kinetics of exogenously administered T cells. However, they did not take into account endogenous T cell homeostasis and its impact on trafficking of the overall T cell population.

PBPK and population PK/PD models, combined with model-based simulations have become a quantitative companion tool in the decision-making process of novel therapies research and development, including small molecule and biologic therapies (25, 26). With the high number of cell-based therapies that are emerging, the development of a validated, flexible framework for PBPK modeling is critically needed (27). Consequently, the main objective of the present work was to develop a general and flexible PBPK framework, whereby, for the first time, quantitative aspects of homeostatic turnover and trafficking of resident T cells, under steady-state conditions, are combined with pharmacokinetic aspects of exogenously administered engineered T cells, within a single PBPK model. Additionally, we aimed at assessing the sensitivity of the model to key parameters controlling T cell migration processes.

## 2 Methods

### 2.1 Experimental data

**Figure 1:**
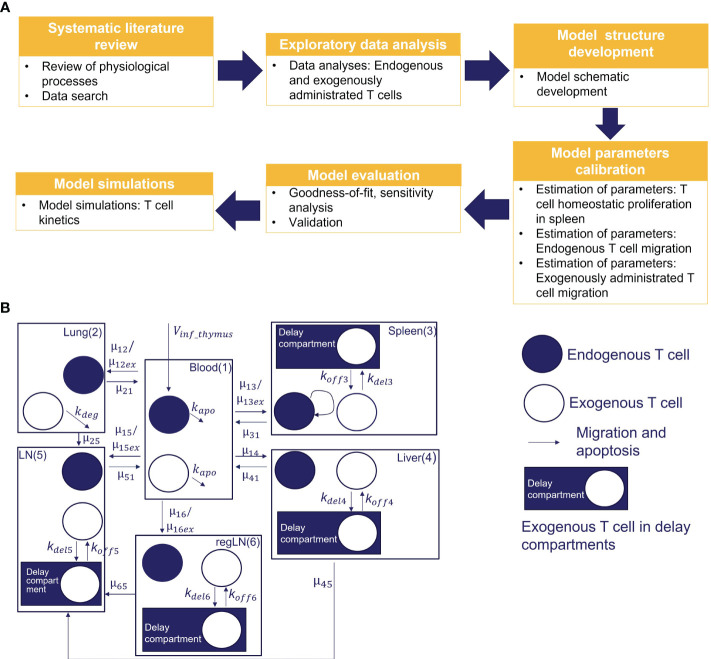
**(A)** Model development workflow; **(B)** Schematic of T cell migration model. The model describes the trafficking of two independent populations of T cells - exogenously administered T cells and endogenous T cells - across 6 compartments (blood, lungs, regional LN compartment, generalized LN compartment, spleen, and liver), with additional intra-tissue, time-related ‘delay compartments’ for exogenously delivered T cells located in the spleen, liver, and the regional and generalized LN compartments.

We conducted a systematic review of published experimental data on T cell trafficking in mouse. Carefully curated datasets were used for model calibration and validation. In total, data from three preclinical studies that included 30 experimental measurements were used for the estimation of model parameters (i.e., 25 values of mean T-cell concentrations in time for different tissues, and 5 values for steady-state concentrations of endogenous T cells). Three additional studies, providing 24 experimental timepoint measurements were used for independent cross-validation. A multistep approach was implemented for model development, as shown in **Figure 1A**.

Parameters of endogenous T cell recirculation were estimated based on experimental data describing T cell concentrations under non-inflammatory conditions and under steady-state physiological conditions in mouse tissues (28, 29). The estimated maximal rate of homeostatic T cell proliferation in the spleen was verified against data in mice which underwent thymectomy (30). Parameters of exogenously administered T cell recirculation were estimated based on murine experimental data from Khot et al. (15). In that study, autologous Cr^51^-labeled spleen T cells were injected intravenously, and radioactivity levels (per gram of tissue) were measured in various organs. To incorporate these data into the model, we parameterized T cell concentrations using **Equation S4** (see **Supplementary Material**). Area-under-the-curve (AUC) estimates show that exogenously administered cells quickly transfer from blood and lung (AUC=(0.089 ± 0.057)* $10^{8}\;$ and (2.26 ± 0.54)* $10^{8}$ cells/ml), to then accumulate in liver and lymph nodes (AUC=(6.75 ± 2.21)* $10^{8}\;$ and (1.62 ± 0.60)* $10^{8}$ cells/ml), to ultimately home to the spleen (AUC= (30.65 ± 11.37)* $10^{8}$ cells/ml).

Experimental data describing T cell kinetics following an intravenous administration of Zr^89^-labeled T/CAR-T cells in mouse were used for an external cross-validation of the model. In those experiments, radioactivity detection was performed via positron-emission tomography (PET) for various types and doses of T cells as follows: (i) infusion of labeled T cells in a dose of 20.0* $10^{6}$ cells (31); (ii) infusion of CAR-T cells in doses of 1.90-, 7.10-, and 16.8* $10^{6}$ cells (16); and (iii) infusion of CAR-T cells in a dose of 1.00* $10^{6}$ cells (32).

### 2.2 Mathematical model

To capture the kinetics and inter-organ recirculation of endogenous and exogenously administered T cells, we developed a physiologically-based compartmental model of T cell migration, formulated with a system of sixteen ODEs. The model scheme is presented in **Figure 1B**. It includes 6 compartments corresponding to the following organs: blood, lungs, spleen, liver, ‘regional LNs’ and ‘generalized LNs’ (the latter representing secondary lymphoid structures). One of the main assumptions in our model relates to the independent circulation of endogenous vs. exogenous T cells in the various organs. Also, we assumed that exogenously administered and endogenous T cells displayed identical rates of organ-to-blood migration. For other model parameters, we considered quantitative differences in parameter values, given that experimental settings of exogenous cell product manufacturing resulted in variously modified T cells. Firstly, we assumed different rates of migration to the spleen, lung and LNs, for endogenous vs. exogenously administered T cells. Secondly, we assumed that the migratory behavior of exogenously administered T cells would not depend on disease status (i.e., identical T cell distribution patterns in healthy vs. tumor-bearing mice, in non-tumor organs). Thirdly, we assumed that exogenously administered T cells undergo degradation in the lungs [via phagocytosis by alveolar macrophages, based on a model by Zhu et al. (33)] and in blood – thereby accounting for all apoptotic processes in the whole organism.

Importantly, we did not include any proliferation potential of exogenous T cells in the model, since we did not consider the mechanisms required to stimulate antigen-specific proliferation (including exogenous T cell activation and interactions with antigen-presenting cells) in the present study.

Model equations were formulated using the following phenomenological view on T cell systemic and tissue dynamics. Endogenous T cells are produced in the thymus and migrate to blood (model parameter *V_inf_thymus_*) and various organs. The rate of homeostatic T cell proliferation in the spleen was described using a Michaelis-Menten like equation. The corresponding EC_50_ value for homeostatic proliferation in the spleen was estimated as the degree of T cell depletion in the mouse spleen following irradiation, upon which proliferating T cells were observed (34). T cells can migrate from blood to tissues and back. For lung and liver compartments, the model considered T cell trafficking to lymph nodes through afferent lymphatic vessels. The rate of T cell migration from LNs to blood was assumed to be the same as the rate of T cell migration through the thoracic duct. Small distal LNs were represented in the model by a regional LN compartment, with T cells being transported from there to a generalized LN compartment via efferent lymphatic vessels.

Overall, intravenously administered T cells were set to follow the same migration patterns as endogenous T cells (see **Figure 1B**). However, characteristic residence times were observed for exogenously administered T cells, in specific organs such as the spleen and the liver (10, 35). Various mechanistic explanations have been put forward to explain these observations; e.g., Ganusov and Tomura proposed that a tight network of sinusoids in liver and spleen may slow down cell migration at supra-high T cell numbers (20). An alternative biological explanation is that the rate of T cell migration may well be heterogeneous and dependent on morphological aspects of the spleen, e.g., the white pulp, red pulp, and marginal zones (22). To integrate these biophysical features into the model, we included additional variables representing the exogenous T cell abundance in the spleen, liver and LNs compartments $(T_{ex\_del\_spleen},T_{ex\_del\_liver},\;T_{ex\_del\_ln},T_{ex\_del\_regln},\;$ respectively). Exogenously administered T cells were set to migrate out of blood and the lungs according to the following clearance processes: systemic apoptosis in blood and degradation by alveolar macrophages in lungs.

The parameters of the model of the model are listed in **Table 1**. Overall, the system of equations was formulated as follows:

**Table 1:** Model parameter values.

Parameter	Value	RSE*	Description	Source
*μ_12_*	2.66 1/h	1.32	Rate constant of T cell migration from blood to lungs	Fitted against (28, 29, 36–38)
*μ_13_*	0.92 1/h	1.42	Rate constant of T cell migration from blood to spleen	Fitted against (28, 29, 36–38)
*μ_14_*	1.05 1/h	1.30	Rate constant of T cell migration from blood to liver	Fitted against (28, 29, 36–38)
*μ_51_*	0.20 1/h		Rate constant of T cell migration from generalized LNs to blood	Calculated based on the rate of daily T cell flow through the thoracic duct (39–42)
*μ_25_*	0.51 1/h	1.70	Rate constant of T cell migration from lungs to generalized LNs	Fitted against (28, 29, 36–38)
*k_apo_*	0.004 1/h	1.00	Rate constant of T cell apoptosis	Fitted against (28, 29, 36–38)
*μ_15_*	2.4 1/h	1.42	Rate constant of T cell migration from blood to generalized LNs	Fitted against (28, 29, 36–38)
*μ_21_*	2.31 1/h		Rate constant of T cell migration from lungs to blood	Calculated based on t_1/2_ in lung from (20)
*μ_31_*	0.17 1/h		Rate constant of T cell migration from spleen to blood	Calculated based on t_1/2_ in spleen from (20)
*μ_41_*	0.63 1/h		Rate constant of T cell migration from liver to blood	Calculated based on t_1/2_ in liver from (20)
V*_inf_*___ *_thymus_*	3.13*10^3^ cells/h		Rate of T cell influx from thymus	Calculated based on (43)
*μ_45_*	0.54 1/h	1.71	Rate constant of T cell migration from liver to generalized LNs	Fitted against (28, 29, 36–38)
*μ_13ex_*	0.064 1/h	10.7	Rate constant of exogenous T cell migration from blood to spleen	Fitted against (15), **Figure 2**
*μ_12ex_*	3.99 1/h	2.19	Rate constant of exogenous T cell migration from blood to lungs	Fitted against (15), **Figure 2**
*k_del3_*	3.55 1/h	24.8	Rate constant of exogenous T cell migration in delay compartment in spleen	Fitted against (15), **Figure 2**
*k_off3_*	0.17 1/h		Rate constant of exogenous T cell migration from delay compartment in spleen	Assumed the same as *μ_31_*
*k_deg_*	0.84 1/h		Constant of T cell degradation in lungs	Fixed based on the value of the elimination rate constant in the model by Khot et al. (15), **Table 2**
*k_del4_*	72.06 1/h	22.2	Rate constant of exogenous T cell migration in delay compartment in liver	Fitted against (15), **Figure 2**
*k_off4_*	0.63 1/h		Rate constant of exogenous T cell migration from delay compartment in liver	Assumed identical to *μ_41_*
*μ_16_*	0.048 1/h	1.42	Rate constant of endogenous T cell migration from blood to regional LNs	Fitted against (28, 29, 36–38)
*μ_16ex_*	0.000086 1/h	13.7	Rate constant of exogenous T cell migration to regional LNs	Fitted against (15), **Figure 2**
*μ_65_*	0.077 1/h		Rate constant of endogenous T cell migration from regional LNs	Calculated based on t_1/2_ in lymph node from (20)
*k_del5_*	3.55 1/h		Rate constant of exogenous T cell migration in delay compartment in generalized LN compartment	Assumed identical to *k_del3_*
*k_off5_*	0.2 1/h		Rate constant of exogenous T cell migration from delay compartment in generalized LN compartment	Assumed identical to *μ_51_*
*k_del6_*	3.55 1/h		Rate constant of exogenous T cell migration in delay compartment in regional LN compartment	Assumed identical to *k_del3_*
*k_off6_*	0.077 1/h		Rate constant of exogenous T cell migration from delay compartment in regional LN compartment	Assumed the same as *μ_61_*
*EC50_pro_*	10^6^ cells		Half-maximal effective number of T cells in terms of proliferation	Assumed based on data on T cell proliferation in irradiated mice (34)
*Vmax_pro_*	12281	1.77	Maximal rate of T cell proliferation in spleen	Fitted against data on thymectomized mice (30)
*f_ccr7_*	0.75		Portion of CCR7^+^ endogenous T cells	Fixed based on experimental data on CCR7^+^ T cells in blood (44)
*f_ccr7_ex_*	0.75		Portion of CCR7^+^ exogenous T cells	Fixed based on experimental data on CCR7^+^ T cells in spleen (44)
V_blood	2.15 ml		Volume of blood, mouse	Taken from (45)
V_lung	0.204 ml		Volume of lungs, mouse	Taken from (45)
V_spleen	0.127 ml		Volume of spleen, mouse	Taken from (45)
V_liver	1.93 ml		Volume of liver, mouse	Taken from (45)
V_ln	0.113 ml		Volume of lymph node, mouse	Taken from (45)
mass_lung	0.130 g		Lung mass, mouse	Taken from (46)
mass_spleen	0.106 g		Spleen mass, mouse	Taken from (46)
mass_liver	1.013 g		Liver mass, mouse	Taken from (46)

*RSE: Relative Standard Error.


(1) $$\begin{array}{c} \frac{dT_{blood}}{dt}=V_{inf\_thymus}-T_{blood}*(\left(1-f_{ccr7}\right)*{µ}_{12}+{µ}_{13}+\left(1-f_{ccr7}\right)\\ {}*{µ}_{14}+f_{ccr7}*{µ}_{15}+f_{ccr7}*{µ}_{16})+{µ}_{21}*T_{lung}+{µ}_{41}\\ {}*T_{liver}+{µ}_{51}*T_{ln}+{µ}_{31}*T_{spleen}-k_{apo}*T_{blood} \end{array}$$

(2) $$\begin{array}{l} \frac{dT_{ex\_blood}}{dt}=-T_{ex\_blood}*(\left(1-f_{ccr7\_ex}\right)*{µ}_{12ex}+\left(1-f_{ccr7\_ex}\right)*{µ}_{14}\\ \;+{µ}_{13ex}+f_{ccr7\_ex}*{µ}_{15ex}+f_{ccr7\_ex}*{µ}_{16ex})+{µ}_{21}\\ \;*T_{ex\_lung}+{µ}_{41}*T_{ex\_liver}+{µ}_{51}*T_{ex\_ln}+{µ}_{31}*T_{ex\_spleen}\\ \;-k_{apo}*T_{ex\_blood} \end{array}$$

(3) $$\frac{dT_{lung}}{dt}=\left(1-f_{ccr7}\right)*{µ}_{12}*T_{blood}-{µ}_{21}*T_{lung}-{µ}_{25}*T_{lung}$$

(4) $$\frac{dT_{ex\_lung}}{dt}=\left(1-f_{ccr7\_ex}\right)*{µ}_{12ex}*T_{ex\_blood}-{µ}_{21}*T_{ex\_lung}-{µ}_{25}*T_{ex\_lung}-\;k_{deg}*T_{ex\_lung}$$

(5) $$\frac{dT_{liver}}{dt}=\left(1-f_{ccr7}\right)*{µ}_{14}*T_{blood}-{µ}_{41}*T_{liver}-{µ}_{45}*T_{liver}$$

(6) $$\begin{array}{c} \frac{dT_{ex\_liver}}{dt}=\left(1-f_{ccr7\_ex}\right)*{µ}_{14}*T_{ex\_blood}-{µ}_{41}*T_{ex\_liver}\\ \;\;-{µ}_{45}*T_{ex\_liver}\;+\;k_{off4}*T_{ex\_del\_liver}\\ \;\;-k_{del4}*T_{ex\_liver} \end{array}$$

(7) $$\frac{dT_{ex\_del\_liver}}{dt}=k_{del4}*T_{ex\_liver}\;-k_{off4}*T_{ex\_del\_liver}$$

(8) $$\frac{dT_{spleen}}{dt}={µ}_{13}*T_{blood}-{µ}_{31}*T_{spleen}+\frac{Vmax_{pro}*T_{spleen}}{EC50_{pro}+T_{spleen}}$$

(9) $$\frac{dT_{ex\_spleen}}{dt}={µ}_{13ex}*T_{ex\_blood}-{µ}_{31}*T_{e\text{x}\_spleen}\;-k_{del3}*T_{e\text{x}\_spleen}\;+k_{off3}*T_{ex\_del\_spleen}$$

(10) $$\frac{dT_{ex\_del\_spleen}}{dt}=k_{del3}*T_{ex\_spleen}-k_{off3}*T_{ex\_del\_spleen}$$

(11) $$\begin{array}{c} \frac{dT_{ex\_ln}}{dt}=f_{ccr7\_ex}*{µ}_{15ex}*T_{ex\_blood\;}+{µ}_{45}*T_{ex\_liver}\\ \;\;+{µ}_{25}*T_{ex\_lung}-{µ}_{51}*T_{ex\_ln}\\ \;\;-k_{del5}*T_{ex\_ln}+k_{off5}*T_{ex\_del\_ln\;}+{µ}_{65}*T_{ex\_regln} \end{array}$$

(12) $$\frac{dT_{ln}}{dt}=f_{ccr7}*{µ}_{15}*T_{blood\;}+{µ}_{45}*T_{liver}+{µ}_{25}*T_{lung}-{µ}_{51}*T_{ln\;}+{µ}_{65}*T_{regln}$$

(13) $$\frac{dT_{regln}}{dt}=f_{ccr7}*{µ}_{16}*T_{blood}-{µ}_{65}*T_{regln\;}$$

(14) $$\frac{dT_{ex\_regln}}{dt}=f_{ccr7\_ex}*{µ}_{16ex}*T_{ex\_blood}-{µ}_{65}*T_{ex\_regln}-k_{del6}*T_{ex\_regln}+k_{off6}*T_{ex\_del\_regln\;}$$

(15) $$\frac{dT_{ex\_del\_ln}}{dt}=k_{del5}*T_{ex\_ln}-k_{off5}*T_{ex\_del\_ln\;}$$

(16) $$\frac{dT_{ex\_del\_regln}}{dt}=k_{del6}*T_{ex\_regln}-k_{off6}*T_{ex\_del\_regln\;}$$

The first equation describes the influx of endogenous T cells from the thymus to the blood compartment (first term: $V_{inf\_thymus}$ ). T cell migration from blood ( $T_{blood})$ to all other compartments (lungs, spleen, liver, generalized LNs and regional LNs with rate constants of, respectively, *µ_12_*, *µ_13_*, *µ_14_*, *µ_15_*, *µ_16_*) is described by the second term. The selective transition of CCR7^+^ T cells to LNs is taken into account by multiplying the rate of T cell migration from blood to LN compartments (regional and generalized) by *f_ccr7_*, and the transfer rate to non-lymphoid organs, where non-naive T cells migrate to, by *(1-f_ccr7_)*. The next five terms in Equation 1 describe T cell migration from, respectively, lungs, liver, spleen, generalized LNs and regional LNs, to blood. The last term of Equation 1 represents the rate of T cell death via apoptosis.

Equation 2 describes the rate of change of exogenously administered T cell abundance in blood, *T_ex_blood_*, following the infusion. This rate is determined by the balance of the rates of cell transitions from blood to lungs, liver, spleen, generalized LNs and regional LNs, and back to blood. The last term in Equation 2 depicts the apoptotic rate of exogenously administered T cells.

Equation 3 describes the rate of change of T cell levels in the lungs. The three terms in Equation 3 represent the kinetics of T cell transition from blood to lungs, from lungs to blood, and from lungs to the generalized LN compartment.

According to Equation 4, exogenously administered T cells migrate to the lungs, and back, via the same route as endogenous T cells. They degrade in the lungs, according to the last term in Equation 4. This T cell degradation feature was taken from the model by Zhu et al. (33).

Equation 5 is similar to Equation 3; it describes the rates of T cell migration from blood to liver and back, and T cell flow within afferent lymphatics to LNs.

Equation 6 represents the migration of exogenously administered T cells from blood to liver and the migration from liver to blood and LNs. The last two terms describe the transitions of exogenously administered T cells through a ‘delay-causing’ compartment within the liver. The rates of T cell inflow and outflow via the delay compartment in Equation 7 are assumed to be linear.

The spleen is a lymphoid organ; we considered T cell homeostatic proliferation, as described by the last term of Equation 8.

Equation 9 depicts processes which affect exogenously administered T cell numbers in the spleen: migration from blood to spleen, and back. To reproduce T cell kinetics in the spleen, another delay compartment was introduced. The last two terms of Equation 9 represent rates of exogenously administered T cell transitions to the delay compartment and back.

Equation 10 describes the transition of exogenously administered T cells within the spleen to the delay compartment and back.

The inflow of CCR7^+^ T cells from blood to LNs through HEVs was taken into account in the first terms of Equations 11, 14 (exogenously administered T cells) and of Equations 12, 13 (endogenous T cells). The first three terms in Equations 11, 12 are similar; they represent the inflow of T cells from blood and the transition of T cells from non-lymphoid organs (liver and lung, respectively). The last terms in these two equations describe the rate of T cell inflow via efferent lymphatics to the generalized LNs. Equation 11 also includes delay-related terms, to capture cell accumulation in the LNs, as shown in previous research (19, 20). The first two terms in Equations 13, 14 represent rates of T cell inflow from blood - through HEVs - to regional LNs and migration via efferent lymphatics to the generalized LNs compartment. For exogenously administered T cells in the regional LN compartment, we also considered cell accumulation via a delay compartment, as described by the last two terms of Equation 14.

Equations 15, 16 represent the dynamics of exogenously administered T cells in the delay compartments for, respectively, the generalized and regional LNs.

Overall, the model includes 30 parameters which are summarized in **Table 1**. Some parameters were assumed to differ between endogenous vs. exogenously administered T cells (e.g., rate constants for migration from blood to spleen and generalized and regional LNs). Parameters which are in common were sub-divided into three groups: a) those verified against data describing steady-state levels of CD3^+^ T cells in mouse tissues (28, 29); b) those estimated based on half-lives of T cells in various organs (19); and c) those estimated using additional experimental data. The last group included parameters for endogenous T cell homeostatic proliferation, which were estimated using data in irradiated mice, and parameters for exogenously administered T cells, which were adjusted against experimental data on exogenously administered T cell kinetics in different tissues (15).

### 2.3 Model calibration

A nonlinear fixed-effect modeling approach was used for the calibration of model parameters with a log-normal distribution assumed for the experimental data. The following log-likelihood cost (LLC) function was maximized to find an optimal set for the fitted parameter values:


$$LL_{y}\left(\hat{\theta }\right)=log\left(L_{y}\left(\hat{\theta }\right)\right)\hat{=}\log(p(y;\hat{\theta }))$$

where $\hat{\theta }$ is the vector of parameter estimates for the model being considered, *y* represents the observed data, and $p(y;\hat{\theta })\;$ is the probability density function of the observed data given the parameter estimates. The model with the highest value of $LL_{y}\left(\hat{\theta }\right)$ was selected as the model of choice.

In addition, relative standard errors (RSE) for model parameters $\hat{\theta }$ and the residual error model were estimated. For all parameters, RSE values lower than 50% were a prerequisite for model selection.

Weighted residuals (WRES) were calculated as the normalized difference between observations and their expected values:


$$WRES=V^{-1/2}\left(y-E\left(\text{y}\right)\right)$$

where  V is the variance-covariance matrix of y , and *E(y)* is the mathematical expectation of the model solutions obtained using parameters $\hat{\theta }$ perturbed by a random value sampled from the residual error model.

### 2.4 Software

The Monolix software, version *2020R1*, was used for fixed-effects modeling, while data visualisation and forward simulations were performed in the *R* software, version *4.0.2* (*R-project*, www.r-project.org). Model sensitivity analyses were performed using the “*sensitivity*” *R* package; model simulations were performed using the “*RsSimulx*” *R* package; visualization of model solutions was performed using “*ggplot2*” and “*tidyverse*” *R* packages.

## 3 Results

### 3.1 Model calibration

We used a sequential approach to curate and integrate all available quantitative information into the model (see the flowchart in **Figure 1A**).

**Figure 2:**
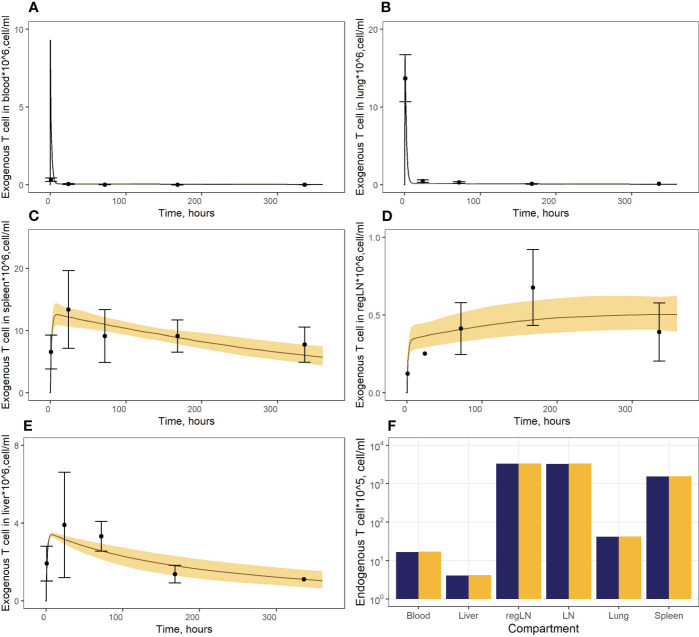
**(A–E)** Model calibration against data on exogenously administered T cells in: **(A)** blood, **(B)** lungs, **(C)** spleen, **(D)** regional LN compartment, and **(E)** liver. Points: Experimental data (mean ± error), calculated from (15). Curves: Model predictions; yellow uncertainty prediction bands: 95% confidence intervals (CI). **(F)** Model calibration against data on endogenous T cells. Dark blue columns: Model predictions; yellow columns: Experimental data, calculated from (28, 29, 36).

In a first stage, we calibrated parameters pertaining to endogenous T cell migration (*V_inf_thymus_*, *µ_12_, µ_13_*, *µ_14_*, *µ_15_*, *µ_25_*, *µ_45_*, *µ_16_*), based on data describing the steady-state levels of T cells in various compartments. In addition, we determined a ratio between rates of T cell migration through HEVs (*V_blood_ln_*) vs. afferent lymphatics (*V_lung_ln_*, *V_liver_ln_*), based on the knowledge that only ∼10% of T cells migrate to lymph nodes via afferent lymphatics (6). The rate constants for T cell migration from various tissues to blood (*µ_21_, µ_31_, µ_41_, µ_61_*) were calculated using estimates of cellular half-lives provided by Ganusov and Tomura (20). The parameter value for *µ_51_* was calculated as the rate of T cell flux to the thoracic duct. As shown in **Figure 2F**, the model consistently described the experimental data, i.e., endogenous T cells would accumulate in lymphoid organs at concentrations of 1.57* $10^{8}\;$ cells/ml in the spleen and 3.36* $10^{8}\;$ cells/ml in LNs, whereas T cell levels in blood and lungs at steady-state were significantly lower (1.70*10^6^ cells/ml and 4.22* $10^{6}$ cells/ml, respectively); and liver exhibited the lowest level of T cell infiltration (∼4.20* $10^{5}$ cells/ml).

**Figure 3:**
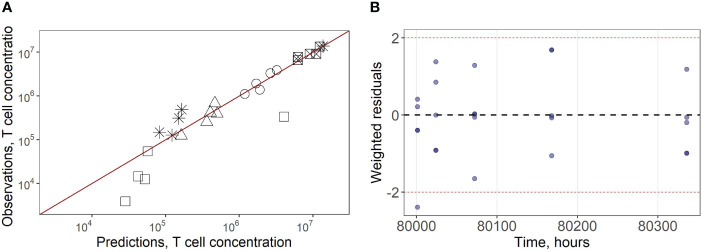
**(A)** Goodness-of-fit for exogenously administered T cell concentrations in spleen (square with a cross inscribed), liver (circles), lungs (asterisks), blood (squares) and regional LN compartment (triangles). **(B)** Weighted residuals for exogenously administered T cells in all compartments.

In a second stage of model calibration, we fixed all parameters for endogenous T cells, all rate constants for exogenous T cell migration from organs to blood (assumed to be the same as for the endogenous cell migration), as well as the parameter that characterizes the rate of T cell apoptosis. We then estimated parameters *µ_13ex_, µ_12ex, _µ_15ex, _µ_16ex_* and parameters describing the delay in T cell migration into liver, spleen and LNs (*k_del3_, k_del4,_ k_del5_, k_del6_*) using experimental data on exogenously administered T cell kinetics (15). Since parameters *k_del3_, k_del5,_ k_del6_* represent the rate constants for the accumulation of cells in lymphoid organs, we assumed the same value for these three parameters. The resultant calibrated model consistently described available experimental data (**Figures 2A–E**). In particular, the model adequately reproduced the fast T cell elimination from blood (**Figure 2A**) and the comparably rapid transitory kinetics in the highly vascularized lungs (**Figure 2B**), as well as the slower kinetics in the spleen (with the peak value *C_max_* of ∼12.6*10^6^ cells/ml at 9 hrs) and the liver (*C_max_* of ∼3.4*10^6^ cells/ml at 8 hrs) (**Figure 2E**). The model also satisfactorily described the cellular kinetics in the regional LN compartment, with a *C_max_* of 0.5*10^6^ cells/ml at 45.5 hrs post administration (**Figure 2D**). Goodness-of-fit plots (**Figure 3**) provide further support for an adequate description of the experimental data by the model. In particular, the moderate over-estimation of *C_max_* in blood holds for only some of the reported blood measurements (squares on **Figure 3A**), with weighted residuals (WRES) lying within a [−2, +2] range (**Figure 3B**).

It is worth noting that, initially, we had considered a model structure featuring only one lumped LN compartment, i.e., without any regional LN compartment (**Supplementary Figure 1**, **Supplementary Material**). The corresponding model could not describe the available experimental LN data for exogenously administered T cells, yet it adequately captured the cellular dynamics in tissues other than LNs (**Supplementary Figure 2**, **Supplementary Material**).

### 3.2 Model validation

To validate the model and assess its predictive power, we performed simulations of external data which had not been used in the model calibration process. In particular, the model was validated against experimental data in mice which received infusions of radiolabeled T cells or CAR-T cells, in doses of 1.0*10^6^, 1.9*10^6^, 7.1*10^6^, or 16.8*10^6^ cells per animal (16, 31, 32). Due to the different methods used for T cell labeling, with corresponding differing detections and units of radioactivity in a region of interest (ROI) in the experimental studies, we adjusted the data using a scaling coefficient, *ω*, in Equation 17 as follows:


(17) C(oncentration) of T cells in organ (scaled){cells/ml} =ω* C of T cells in organ{cells/ml}

An averaged calibration coefficient value of 2.7 was determined for *ω*, although there are several reasons for significant variability across studies due to the characteristics of the various experiments (See Section 5, **Supplementary Material**).

**Figure 4:**
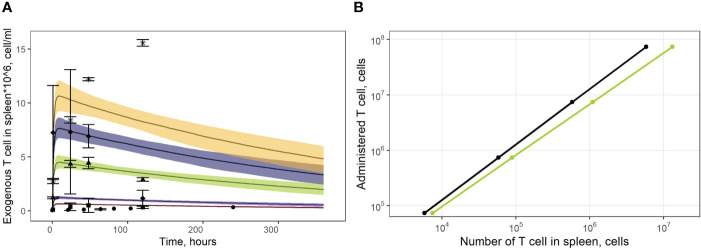
**(A)** Model validation against data on exogenously administered, radiolabeled T/CAR-T cells in mouse spleen. Solid curves: model predictions; bands: 95% confidence intervals; points: experimental data with confidence intervals taken from publications of Sta Maria et al. and Wang et al. (16, 31, 32). Brown shadows and datapoint circles correspond to the administration of CAR-T cells (10^6^ cell dose); violet shadows and datapoint squares: administration of CAR-T cells (1.9*10^6^ cell dose); green shadows and datapoint triangles: administration of CAR-T cells (7.1*10^6^ cell dose); dark blue shadows and datapoint rhombi: administration of T cells (12.0*10^6^ cell dose); yellow shadows and datapoint asterisks: administration of CAR-T cells (16.8*10^6^ cell dose). **(B)** Dependence of exogenously administered T cell numbers in the spleen vs. given dose of labeled T cells. Black line: model simulations; green line: experimental data taken from (13).

As shown in **Figure 4A**, the model adequately described experimental data for doses of 1.9*10^6^ and 7.1*10^6^ CAR-T cells. However, it slightly over-predicted peak levels following doses of 1.0*10^6^ and 1.9*10^6^ (*C_max_* values of 0.6*10^6^ and 1.1*10^6^ cells/ml predicted by the model vs. observed *C_max_* values of 0.3*10^6^ and 0.5*10^6^ cells/ml, respectively). At the same time, T cell levels at later timepoints, following a dose of 16.8*10^6^ cells were under-estimated, with a predicted value *C_max_* of 1.02*10^7^ cells/ml vs. a higher *C_max_* of 1.50*10^7^ cells/ml observed in the experimental data. These differences between observed and model-predicted values may have been caused by uncertainties related to the calibration of the radioactivity labeling data. Since the actual calibration curves for the various experimental settings were not available, we applied an empirical approach to map the data onto a uniform scale, which may have introduced an additional bias. To further validate the model, we also used external data on exogenous T cell levels in the spleen, under various doses (13). The linear dependence between T cell counts in the spleen and the administered doses was successfully reproduced by the model, for this external set of experimental data (**Figure 4B**).

### 3.3 Sensitivity analysis

Two types of sensitivity analyses were conducted: a global sensitivity analysis for parameters and variables describing endogenous T cell homeostasis, and a local sensitivity analysis for those describing exogenously administered T cell numbers and accumulation.

The Partial Rank Correlation Coefficient (PRCC) method was used to perform the global sensitivity assessment of the model (47). Model parameters were sampled using a Latin hypercube sampling (LHS) procedure. 5000 sets of parameters were sampled for single estimation of PRCC. The procedure was repeated 200 times to estimate standard errors. The lower and upper bounds of the parameters were assumed to be determined by values corresponding to a two-fold change from steady-state T cell concentrations. Model solutions were computed over a large time window (up to 80,000 hrs), to ensure steady-state conditions. Under such a setting, the PRCC may characterize the relative importance or effect of a parameter, along with its positive/negative correlation mode, vs. model output variability.

**Figure 5:**
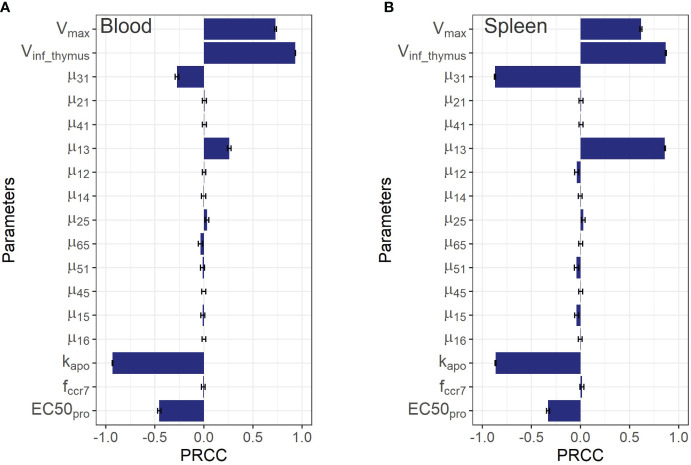
Global sensitivity analysis for steady-state concentrations of endogenous T cells in: **(A)** blood; and **(B)** spleen. Bars depict PRCCs (partial rank correlation coefficients) for each parameter (mean ± standard error).

As shown in **Figure 5**, the PRCC coefficients for the rate constants of T cell migration from blood to spleen and back (*µ_13_* and *µ_31_*) were about 4-times higher in terms of their impact on steady-state levels of T cells in spleen vs. blood. A similar relationship was found for the lungs (see **Supplementary Figure 3** in the **Supplementary Material**): the rate constants of migration from blood to the lungs and back (*µ_12 _* and *µ_21_*) were more important for the lungs than for blood, with corresponding PRCC coefficients about 10-times higher. According to this sensitivity analysis, the key systemic parameters with comparable impact on T cell homeostasis in all compartments were *V_inf_thymus_* and *k__apo_*. Parameters of homeostatic proliferation *Vmax_pro_* and *EC50_pro_* were also important for T cell levels in all compartments.

Although the sensitivity analysis predicted rather low PRCC coefficients for T cell inflow rate parameters from the lungs and liver to the LNs via afferent lymphatic vessels (*µ_25_* and *µ_45_*), in relation to T cell levels in the blood and the spleen, it revealed that these processes were essential in driving T cell levels in the organs themselves. This is demonstrated by the 5x higher values of the corresponding PRCC coefficients for the lungs and liver vs. other compartments (excluding LNs for *µ_25_*), which highlights the importance of considering the model’s sensitivity to the lymphatic system parameters. The T cell inflow constant from lungs to LNs, *µ_25_*, carried significance for homeostasis in the lung and LNs, as shown in **Supplementary Figures 3A, C** of the **Supplementary Material**. Also, it should be noted that the parameter $f_{ccr7}$ was predicted to play a minor influence on T cell homeostasis in blood and spleen, while the levels of T cells in liver, the lungs, and both regional and generalized LN compartments appeared to be more sensitive to $f_{ccr7}$ .

**Figure 6:**
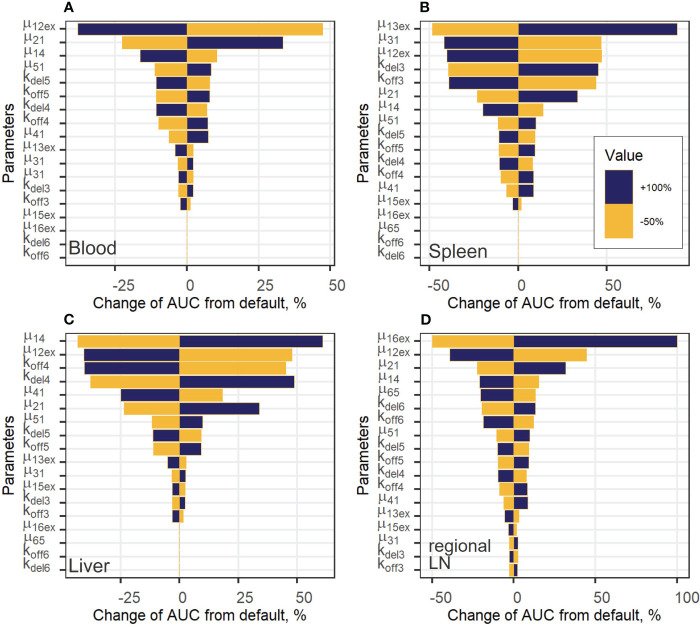
Local sensitivity analysis for exogenous T cell steady-state concentrations in: **(A)** blood; **(B)** spleen; **(C)** liver; and **(D)** regional LN compartment.

To estimate the impact of kinetic parameters on exogenously administered T cell dynamics, a local sensitivity analysis was conducted. Parameter values were varied in the following way: decreased by 50% or increased by 100% from their best-fit estimates (i.e., two-fold changes). **Figure 6** shows the sensitivity profiles for exogenous T cell in blood (A), spleen (B), liver (C) and regional LN (D). **Supplementary Figure 6** in the **Supplementary Material** presents the dependence of the total number of exogenous T cells vs. time, which was calculated as the sum of T cell numbers in all compartments. For all compartments excluding blood, the most important parameters were the rate constants of T cell inflow from blood to organ: changes in *µ_13ex_, µ_14_* and *µ_16ex_* resulted in proportional changes of the AUC in spleen, liver, and regional LNs, respectively. The same behavior was shown to hold for the lungs (see **Supplementary Figure 4A** in the **Supplementary Material**).

For the generalized LN compartment (see **Figure S4B**), the most important parameter was the T cell inflow rate from organ to blood (*µ_51_*). Interestingly, the rate constant of T cell transition to the lungs, *µ_12ex_*, was significant with respect to all variables, including the level of T cells in blood. This rate relates to the change in the fraction of total exogenous T cells trafficking to the lungs, where a relatively high level of T cell degradation is set in the model (**Supplementary Figure 6B**), red and grey curves, **Supplementary Material**). Also, an increase in the rate constant, *k_del3_*, which describes the T cell transition rate to the delay compartment in the spleen, resulted in a stronger accumulation of exogenous T lymphocytes in the delay compartment and a higher AUC in the spleen (**Figure 6B**). The same sensitivity pattern was observed for other delay compartments: increases in *k_del4_, k_del5_* and, to a lesser degree, in *k_del6_* resulted in higher T-cell AUC values in the liver as well as in the generalized LN and regional LN compartments. On the contrary, increases in *k_off_* parameters resulted in a faster migration to, and exit from the corresponding compartments and, as a consequence, in lower AUC values (as shown for *k_off3_*, *k_off4_*, *k_off6_* in **Figures 6B–D**), respectively). The rate constant capturing the inflow of cells to the regional LN compartment and the delay-related parameter for that compartment (*µ_16ex_*, *k_del6_*) were important only within this compartment; inflow rate constants and delay parameters in all other compartments had a low impact on T cell AUC values. We also explored the importance of these parameters with respect to their effects on *C_max_* values (**Supplementary Figure 5**, **Supplementary Material**) and obtained similar results. Except for parameters *µ_12ex,_ µ_13ex,_ µ_14,_ µ_16ex_*, which were the most important parameters determining *C_max_* in the corresponding compartments, the inflow rates from blood to other compartments were more significant than the rate constants reflecting transitions to delay compartments. For example, for the value of *C_max_* in the spleen, the rate constants of T cell inflow to the lungs and liver (*µ_12ex_*, *µ_14_*) were more significant than *k_del3_*, *k_off3_*.

Overall, these kinetic behaviors reflect the inter-dependence of T cell levels in various organ and tissue compartments, which are inherently linked in a continuous flow-like system such as the whole organism.

### 3.4 Model simulations

**Figure 7:**
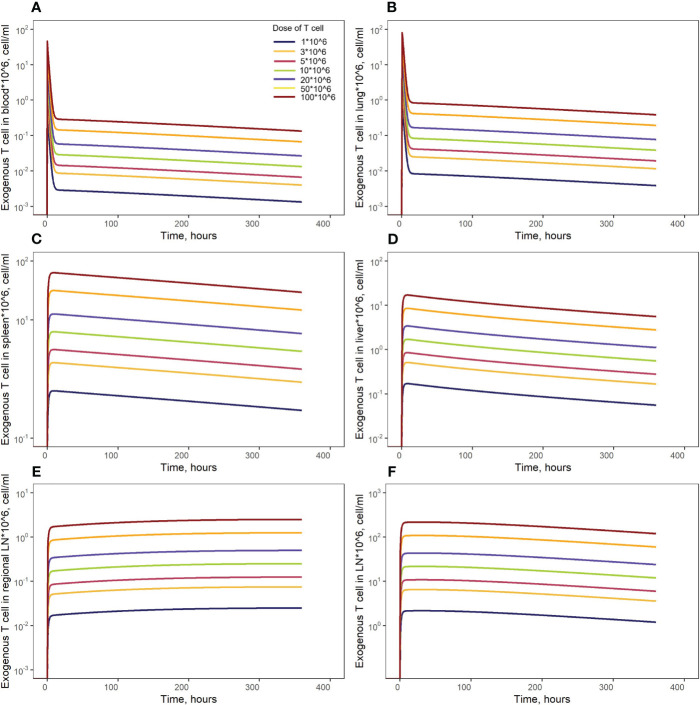
Model simulations of exogenously administered T cell recirculation kinetics upon given cell doses of 1.00*10^6^ to 100*10^6^ cells in: **(A)** blood; **(B)** lungs; **(C)** spleen; **(D)** liver; **(E)** generalized LN compartment; and **(F)** regional LN compartment.

Model simulations were conducted to investigate the effects of specific factors such as exogenous T cell dose and CCR7 expression levels, on T cell kinetics and distribution. To explore more specifically the dose dependency on cellular kinetics, we performed the following numerical experiments representing intravenously administered T cells at doses of 1.0, 3.0, 5.0, 10, 20, 50, or 100 million cells per mouse (**Figure 7**). Low doses of 1.0, 3.0, 5.0 or 10 million T cells resulted in *C_max_* values in blood of, respectively: {0.46, 1.40, 2.30, 4.65}*10^6^ cells/ml. High doses of 20, 50, or 100 million T cells resulted in *C_max_* values of, respectively, {9.30, 23.3, 46.5}*10^6^ cells/ml. Consequently, there is a linear relationship between the dose of adoptively transferred cells and T cell concentrations in the spleen (see **Figure 4B**, 72 hr post-infusion timepoint), which is observed experimentally and predicted by the calibrated model. A similar relationship was revealed in other compartments (see **Figures 7B–F**). We also simulated T cell concentration in the generalized LN compartment (**Figure 7F**) and found a linear dependence between the dose and the accumulation of lymphocytes in this compartment.

**Figure 8:**
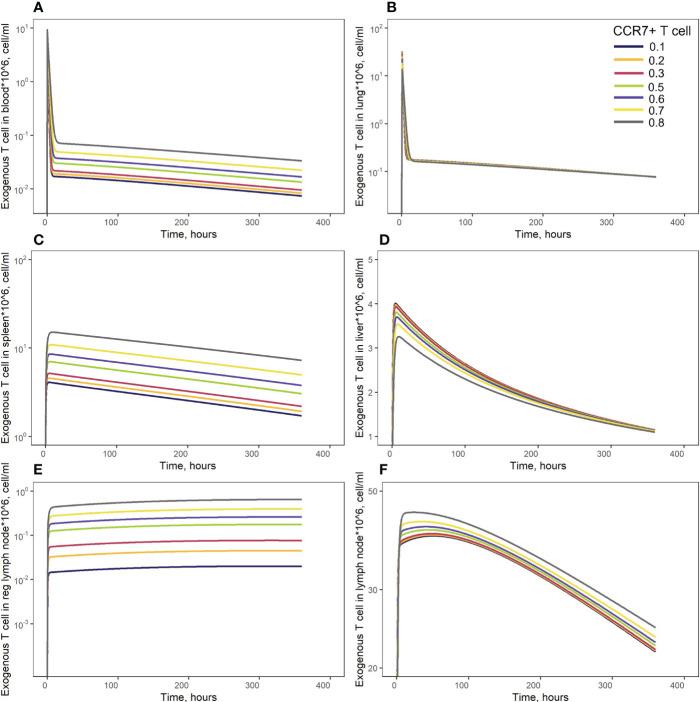
Model simulations of exogenous T cell recirculation kinetics following an administration of T cells in doses of 20 million cells with different CCR7^+^ T cell proportions (0.1 - 0.9), **(A)** blood; **(B)** lungs; **(C)** spleen; **(D)** liver; **(E)** generalized LN compartment; and regional LN compartment **(F)**.

In order to investigate distribution effects of CCR7 expression levels on exogenously administered T cells, the parameter *f_ccr7_ex_* was varied over a range of 0.1 to 0.9 (**Figure 8**). As summarized in **Table 2**, a change in *f_ccr7_ex_* from 0.1 to 0.3 did not significantly affect the exogenous cell AUC in spleen and blood (respectively, {9.91,11.09,12.59}*10^8^; and {0.11, 0.12, 0.14}*10^8^ cells/ml). A further increase in CCR7 expression levels (with increasing *f_ccr7_ex_* values of 0.7, 0.8, 0.9) led to a higher accumulation of T cells in spleen and blood, with AUC levels of {27.52, 39.1, 67.38}*10^8^ in the spleen and {0.26, 0.36,0.61}*10^8^ in blood (**Figure 8C**). Interestingly, this non-linear relationship is not observed in the liver and lung compartments, where the effects of varying CCR7 expression levels were less pronounced. Indeed, there were only minor differences in kinetics, whether low (0.1 to 0.5) or high (0.7 to 0.9) values of *f_ccr7_ex_* were tested (**Figures 8B, D**). The *C_max_* value in non-lymphoid organs, reflective of the distribution phase, was inversely related to *f_ccr7_ex_*: it increased from 2.63*10^6^ up to 4.01*10^6^ cells/ml for the liver, and from 7.67*10^6^ to 31.3*10^6^ cells/ml for the lungs, with decreasing values of *f_ccr7_ex_* (from 0.9 to 0.1). However, during the elimination phase, higher *C_max_* values led to a slightly lower trough concentrations in the lungs at 360 hrs, with trough cell levels decreasing from 7.7*10^4^ to 7.5*10^4^ cells/ml. Therefore, the lowest value of *C_max_* (*f_ccr7_ex_* = 0.9) led to the highest trough level of T cells in the lungs, although the difference was rather modest.

**Table 2:** Area-under-the-curve (AUC) values and biodistribution coefficients (BC) as a function of CCR7^+^ cell proportions (*f_ccr7_ex_*) in exogenously administered T cells.

AUC** Spleen	AUC** Lung	AUC** Blood	AUC** Liver	AUC** ‘gen’ LNs	AUC** ‘reg’ LN	BC*** Lung	BC*** Spleen	BC*** Liver	BC*** ‘gen’ LNs	BC*** ‘reg’ LN	f_ccr7_ex_
9.906	0.807	0.114	7.827	117.110	0.067	7.079	86.89	68.658	1027.281	0.588	0.1
11.089	0.809	0.123	7.789	117.614	0.149	6.577	90.154	63.325	956.211	1.211	0.2
12.594	0.810	0.135	7.741	118.254	0.254	6.0	93.290	57.341	875.956	1.881	0.3
17.281	0.807	0.174	7.591	120.251	0.581	4.638	99.316	43.626	691.098	3.339	0.5
27.521	0.788	0.260	7.260	124.609	1.291	3.031	105.85	27.923	479.265	4.965	0.7
39.095	0.756	0.359	6.883	129.528	2.089	2.106	108.90	19.173	360.802	5.819	0.8
67.376	0.666	0.606	5.945	141.526	4.017	1.10	111.18	9.810	233.541	6.623	0.9

‘gen’ LNs: generalized LN compartment; ‘reg’ LNs: regional LN compartment.

**AUC values in cells/ml*10^8^.

***BC is a biodistribution coefficient, calculated as the AUC_(organ)_-to-AUC_(blood)_ ratio.

This observed non-linearity in cell kinetics also led to significant differences in the biodistribution coefficient, *BC* (**Table 2**). An increase in *f_ccr7_ex_* resulted in an increase in *BC* in the regional LN compartment, yet in a reduced T cell concentration relatively to blood in the generalized LN compartment (with a lower *BC* value in LNs). *BC* in non-lymphoid organs was also diminished; an increase in $f_{ccr7\_ex}\;$ resulted in a higher *BC* in the spleen compartment. Interestingly, the AUC value appeared to change faster in blood vs. LNs.

To illustrate key differences in the kinetic behavior of exogenous vs. endogenous T cell populations, we performed simulations which simultaneously tracked the kinetics of these two T cell populations. Concentrations of endogenous T cells were at steady-state in each compartment (see **Figure 2F** and **Supplementary Figure 7**, dark red lines, **Supplementary Material**). Since exogenous T cells were administered in high numbers, their kinetics appeared as temporary ‘perturbations’ in T cell numbers in various compartments, with higher or lower exposures depending on the presence of delay compartments (see **Figures 2A–E**) and **Supplementary Figure 7**, dark red lines, **Supplementary Material**). However, even the largest dose of exogenously administered T cells would not exceed ∼1% of the total lymphocyte count in mouse; exogenous T cells would therefore not be expected to significantly perturb the dynamics of endogenous T cells.

## 4 Discussion

Various approaches have been used to study T cell migration and distribution processes in the body: experimental methods include the use of exogenously administered T cells (labeled, pre-activated, with subsequent antigen injection, etc.), whereas theoretical approaches include the development and analysis of mathematical models describing T cell migration throughout the whole organism and within individual organs. The works by Mohler and Farooqi (18), Ganusov and Auerbach (19) and others have yielded significant insights into the kinetics of T lymphocyte migration and distribution. However, given the growing interest in both fundamental immunology and practical applications of preclinical and clinical research, there is a pressing need for an analytical tool that can be used to quantitatively predict proportions of T cells which may enter a target organ in response to, e.g., adoptive T cell therapy or immune activation. Therefore, the main objective of our work was to characterize, via modeling, the migratory behavior and inter-compartmental trafficking of T cells, under homeostatic conditions and upon intravenous infusion.

We thus developed a physiologically-based compartmental model describing the dynamics of exogenously administered T cells and endogenous T cells. The model was used to quantitatively capture homeostatic levels of T cells in mouse tissues and the recirculation kinetics of exogenously administered T cells following intravenous infusion. The model includes time-dependent variables representing CD4^+^ and CD8^+^ T lymphocytes as a single population of CD3^+^ T cells, even though it is known that CD4^+^ and CD8^+^ T cells differ in their rates of migration, apoptosis, and proliferation (48). It is established that the expression of CCR7 is a key determinant for the migration of T lymphocytes to the LNs via HEVs (6). We considered this feature by including parameters *f_ccr7_* and *f_ccr7_ex_* into the model, which represent the fractions of CCR7^+^ T cells in the populations of endogenous and exogenously administered T lymphocytes, respectively. In the model, the entry of T cells into the regional and generalized LN compartments was CCR7-dependent. We neglected any potential effects of CCR7 expression on the inflow to the spleen, in agreement with experimental data showing that mainly CCR7^-^ T cells were detectable in the spleen (49). However, this receptor is significant for further migration through the spleen to zones (e.g., white pulp) where active immune responses take place and may, therefore, be important when modeling T cell activation processes in the spleen.

Model parameters were verified based on data from exogenously administered T cell studies.

In an initial version of the model, only one generalized LN compartment was included. The model adequately reproduced T cell kinetics in all compartments with the exception of the LNs, where the model solution predicted higher levels of T cells. The main reason for this discrepancy lied in the structural difference between our model-based description of LNs, as a lumped compartment, vs. distinct LNs with specific features underlying the experimental data. In that initial model, the lumped LN compartment would collect T cells in the lymph from a number of organs (spleen and lungs), with a high rate of efflux into blood (as calculated from the rate of T cell flux in the thoracic duct). In contrast, the experimental data described T cell kinetics in distinct, specific lymph nodes, which collect T cells from the mouse’s hind leg. With the addition of a regional LN compartment representative of these smaller, distal LNs to the system of model compartments, we were able to consistently describe the experimental data. At the same time, the generalized LN compartment provided an opportunity to estimate the inflow of T cells from the lymphatic system to blood more adequately.

In order to assess the predictive power of the model, we used experimental data on Cr^51^-labeled T cell distribution (15, 16, 31), since Cr^51^ labeling is a broadly used measurement technique in T cell distribution studies. More recent studies have also been conducted, with an estimation of T cell radioactivity using PET measurements in the live mouse (16, 31, 32). In the absence of calibration curves across the various radioactivity measurement experiments, we had to introduce an empirical scaling factor, *ω*, into the model, to map the data from these heterogeneous studies onto a common scale, although this may have introduced an inaccuracy in the dose vs. T cell number relationship. Our mathematical model adequately described the T cell recirculation kinetics following low and intermediate doses of administered CAR-T/T cells. However, for the highest administered dose, the model performed well only during an early time window following cell infusion (first 48 hours). There may be two reasons to explain such discrepancy: (i) a non-linear relationship between the dose (absolute number of T cells) and radioactivity may be in effect, as has been shown using another radioactive label (17); and (ii) altered intrinsic properties of CAR-T cells engineered ex vivo. In the Sta Maria et al. study, mice were infused with CAR-T cells, whereby the CAR included a 41-BB co-receptor (16). There is indirect evidence indicating that persistence of CAR-T with 41-BB may differ vs. other T/CAR-T cells (50).

We also conducted a global sensitivity analysis to rank the mathematical model parameters with respect to their impact on changes in endogenous T cell dynamics in tissues. We determined that the rates of influx and efflux from tissues were more important for T cell homeostasis in organs than in blood. Systemic parameters affected homeostatic T cell levels in blood, lymphoid and non-lymphoid tissues. It is known that only ∼10% of T cells reach LNs via afferent lymph vessels. The mathematical model shows that this flow is a more important determinant of homeostatic levels of T cells in organs from which lymphocytes were released vs. the steady-state level of T cells in LNs (6). The parameter *f_ccr7_*, which captures the proportion of CCR7^+^ endogenous T cells, had a higher influence on T cell levels in non-lymphoid organs and in regional LNs vs. in the spleen, generalized LNs, or blood. The local sensitivity analysis we performed for exogenous T cells also showed the importance of the rates of influx and efflux from tissues. Importantly, the rate of inflow to the lungs also had a strong effect on exogenous T cell accumulation. In addition, the maximal level of T cells (*C_max_* value) depended on rates of influx into other organs, reflective of a continuous network-type and flow-like system.

Using the model, we simulated T cell kinetics and distribution following an intravenous administration of various doses of T cells engineered ex vivo. The model predicted a linear dose-dependence in blood and spleen, in full accordance with results reported by Zatz et al. (13). We conducted further simulations of engineered T cell infusions with differing fractions of CCR7^+^ T cells. Increasing the fraction of CCR7^+^ T cells in the infused dose contributed to an increase in T cell numbers in the spleen. This is supported by indirect evidence from the literature: when CAR-T cells were preincubated with IL-7/IL-15, CCR7^+^ (TCM, TSCM) cell counts in the infusate subsequently increased (51), and IL-7/IL-15-preincubated CAR-T cells were present in higher numbers in the spleen, 3 days post-infusion (52). While CCR7 expression may be associated with cytotoxic activity, cytokine production, and proliferation potential, its effect on migratory features of T cells has already been exploited in CAR-T development.

Our modeling study also presents some limitations. Firstly, we assumed that trafficking of endogenous vs. exogenously administered T cell populations would occur independently of one another. These two subsets of T cells, however, share similar cytokine/chemokine receptors and could, for example, compete for corresponding ligand molecules or interact with each other while migrating through the same vessels and tissues. Also, these two T cell populations interact during *in vivo* expansion and functional cytotoxic responses. However, including such exogenous vs. endogenous T cell interaction processes would require direct experimental data. Additional experimental data would be needed for the quantitative introduction and explicit inclusion of various T cell sub-populations (e.g., CD4^+^ and CD8^+^ T cells) into the model. Such an extension would be warranted to take into account processes related to antigen stimulation, whereby distinct T cell sub-populations would need to be described separately (proliferating/non-proliferating, naïve, effector, memory, resident T cells). Also, we did not consider the proliferation of exogenous T cells in this model, since mechanisms of exogenous T cell activation and interactions with antigen-presenting cells were not included in the present study; experimental data (Cr^51^-radiolabeled T cells) used for model calibration did not include quantitative information on newly developing T cells. Despite such limitations, the quantitative multi-compartmental PBPK model presented here holds the potential for integration into a broader multiscale immune system model. It may be further used to address questions underlying the rational development of immune cell-based therapies and anti-infection therapies of an immunological nature.

The model may also be applied, preclinically, to perform predictive simulations of genetically modified T lymphocyte levels in various organs, following the administration of adoptive T/CAR-T cells. In recent years, various gene modifications of autologous T cells (‘armored’ T cells) have been developed and tested in preclinical and clinical studies (53). The effects of such modifications, e.g., on T cell persistence, may be captured using the model structure presented here, based on changes in the parameter $k_{apo}$ . Gene modifications resulting in changes in cell migration (e.g., CCL19 expression by CAR-T cells) could be investigated by adding a tumor compartment in the model and assuming an ‘LN-like’ influx rate into such a compartment, with CCL19-producing CAR-T cells and all other CCR7^+^ T cells (exogenous and endogenous) migrating, to some degree, to the same locations.

One further application of this model relates to its translation to human studies, e.g., to address the kinetics of immune cells distribution throughout the human body to complement a recent systematic analysis presented in (36). Using human data on T cell homeostasis and dynamics of exogenously administered T cells, we may scale parameters from the present model, calibrated for mouse, to derive a corresponding model for human. Such a model may then allow for predictive simulations of cell kinetics following adoptive T/CAR-T cell therapies, an effective tool for dose optimization purposes with such therapeutic approaches.

## Data availability statement

The original contributions presented in the study are included in the article/**Supplementary Materials**, further inquiries can be directed to the corresponding author/s.

## Author contributions

AN: Investigation, Writing – original draft, Visualization. GH: Validation, Writing – review & editing. KP: Conceptualization, Supervision, Validation, Writing – review & editing. GB: Conceptualization, Supervision, Validation, Writing – review & editing.
