# Supplementary material for: Mathematical modeling of endogenous and exogenously administered T cell recirculation in mouse and its application to pharmacokinetic studies of cell therapies
Source: Front Immunol. 2024 Apr 17;15:1357706. doi: 10.3389/fimmu.2024.1357706 (PMC11155669; doi:10.3389/fimmu.2024.1357706)
Supplement: Supplementary file 1 [file DataSheet_1.docx]

Supplementary Material

**Mathematical modeling of endogenous and exogenously administered T cell recirculation in mouse and its application to pharmacokinetic studies of cell therapies.**

**Antonina Nikitich^*^; Gabriel Helmlinger, PhD; Kirill Peskov, PhD; Gennady Bocharov, PhD**

*** Correspondence:** Antonina Nikitich: [nikitich_a_a@staff.sechenov.ru](mailto:nikitich_a_a@staff.sechenov.ru)

# Model calibration against data on steady-state levels of T cells in organs

For the calibration of parameters describing endogenous T cell migration under homeostatic conditions, we used literature data on T cell levels in organs (Table S1) and fitted the model against these data using the *Monolix* software, version *2020R1*. Also, for model calibration, we used the ratio of rates of T cell migration through HEVs (V*_blood_ln_*) vs. afferent lymphatics (V*_lung_ln_*, V*_liver_ln_*), which are captured in the model by the following equations:

V*_blood_ln_*${=f}_{ccr7}*\mu_{15}*$T*_blood_* (S1)

V*_lung_ln_*=${(1-f}_{ccr7})*\mu_{25}*$T*_lung_* (S2)

V*_liver_ln_*=${(1-f}_{ccr7})*\mu_{45}*$T*_liver_* (S3)

Is is known that ~10% of all T cells in LNs come from afferent lymphatic vessels. In the present model, we lumped lymph nodes into two categories of lymph nodes: a ‘generalized LN’ compartment, with T cells from the lungs and the liver migrating into that compartment; and a ‘regional LN’ compartment, representative of distal LNs. We assumed that, for each influx rate, 5% of all T cells would migrate to the generalized LN compartment. Hence, the V*_blood_ln_*-to-V*_lung_ln_* ratio was calculated as 100:5 = 20. This value was directly used for model calibration.

# Modeling of homeostatic proliferation

We described the rate of homeostatic proliferation using a Michaelis-Menten type equation. It is known that, in mouse, T cells in the spleen proliferate, following radiation-induced decreases in T cells. We determined levels of T cells in the spleen following a 600 rad dose exposure in mouse. It has been shown that, when T cell levels were decreased to ~10^6^ cells, the rate of proliferation was non-zero (1). We thus assumed an EC_50_ value for homeostatic proliferation of 10^6^ cells. Using data on thymectomized mice (2), we developed a submodel under the *Monolix* software, version *2020R1* and verified this parameter for a maximal proliferation rate.

# Calculation of T cell influx from the thymus

It is known that ~1% of thymic cells are involved in the daily thymic output. The total number of thymocytes is 7.5*10^6^ cells (see Reference (3) and Figure 3 (D)). Hence, the influx of T cells from the thymus was taken as 7.5*10^4^ cells/day or 3.13*10^3^ cells/h.

# Calculation of absolute T cell concentration values

In the published references we used, data on T cell distribution were provided in “*ID/g*” (percentage of injected dose per gram tissue). We mapped such data to concentration values using Equation S4, per organ of interest:

$C\mathrm{of}T cell in organ \{cells/ml\}= ID/g * weight of organ*\frac{Dose}{Volume of organ}$ (S4) (see also Table S1)

The values of weights and volumes of organs used in the model are summarized in Table 1 of the Main Manuscript.

# Detection features and units of radioactivity in a region of interest (ROI) in the experimental studies

Various radionuclides are used to visualize T cell distribution, e.g., Zr^89^, Cr^51^, Ga^68^. These labels exhibit different half-lives in the organism, as well as radioactivity levels. Furthermore, even in studies making use of one unique label, inter-individual variability can be rather high. In studies with Zr^89^-radiolabeled CAR-T cells in vitro, the adsorbed dose per a given number of cells was measured (4). The researchers then estimated the adsorbed dose (in *kBq*) out of the injected dose and used it to calculate the absolute number of injected T cells, assuming a linear dose-radioactivity dependence. In another study examining Zr^89^-T cell administration in mouse, the authors estimated the specific activity (in *µCi*) per 10^6^ cells and also used a linear calibration curve (5). However, in yet other studies, the dose-radioactivity dependence curve exhibited some level of saturation, as opposed to plain linearity (6). In addition, experiment protocols may vary in terms of level of radioactivity per cell; it is therefore not possible to use the same calibration curve across two different experiments. Thus, in the absence of specific dose-radioactivity calibration curves, it becomes problematic to properly estimate the absolute number of cells from studies making use of radiolabeled lymphocytes. Even though a precise quantitative estimation of T cell levels is often not the primary aim of empirical studies with radiolabeled cells, such data are required for correct model comparison vs. experimental data.

A further source of variability related to the abovementioned experimental approach pertains to the actual radioactivity detection technology. For example, PET (positron-emission tomography) vs. gamma counter may be used to measure radioactivity levels. In the study by Khot et al. (7), a gamma counter was used, while other studies used PET.

Overall, to include one general measure of radioactivity variability in the model, we considered a calibration or scaling coefficient, *ω*.

**Table S1.** Values of T cell steady-state concentrations in organs (mouse data).

| Parameter | Value | Unit | Description | Reference |
| --- | --- | --- | --- | --- |
| C*_blood_* | 1.7*10^6^ | cells/ml | Concentration of T cells in blood | (8), Fig.1(I) |
| C*_lung_* | 4.22*10^6^ | cells/ml | Concentration of T cells in lungs | (9,10) Fig.1(B) and Fig.1(G), respectively |
| C*_spleen_* | 1.57*10^8^ | cells/ml | Concentration of T cells in spleen | (11), Table 1 |
| C*_liver_* | 4.2*10^5^ | cells/ml | Concentration of T cells in liver | (12), Fig.1(J) |
| C*_ln_* | 3.36*10^8^ | cells/ml | Concentration of T cells in lymph nodes | (8,10), Fig.1(K), Supplementary2, respectively |

**Figure S1.** Scheme of the initial model version with only one lumped LN compartment, referred to as the ‘generalized LN’ compartment. The model describes the trafficking of two independent populations of T cells - exogenously administered T cells and endogenous T cells - across five compartments (blood, lungs, one lumped LN compartment, spleen, and liver), with additional ‘delay compartments’ for exogenously delivered T cells, located in the spleen and the liver.
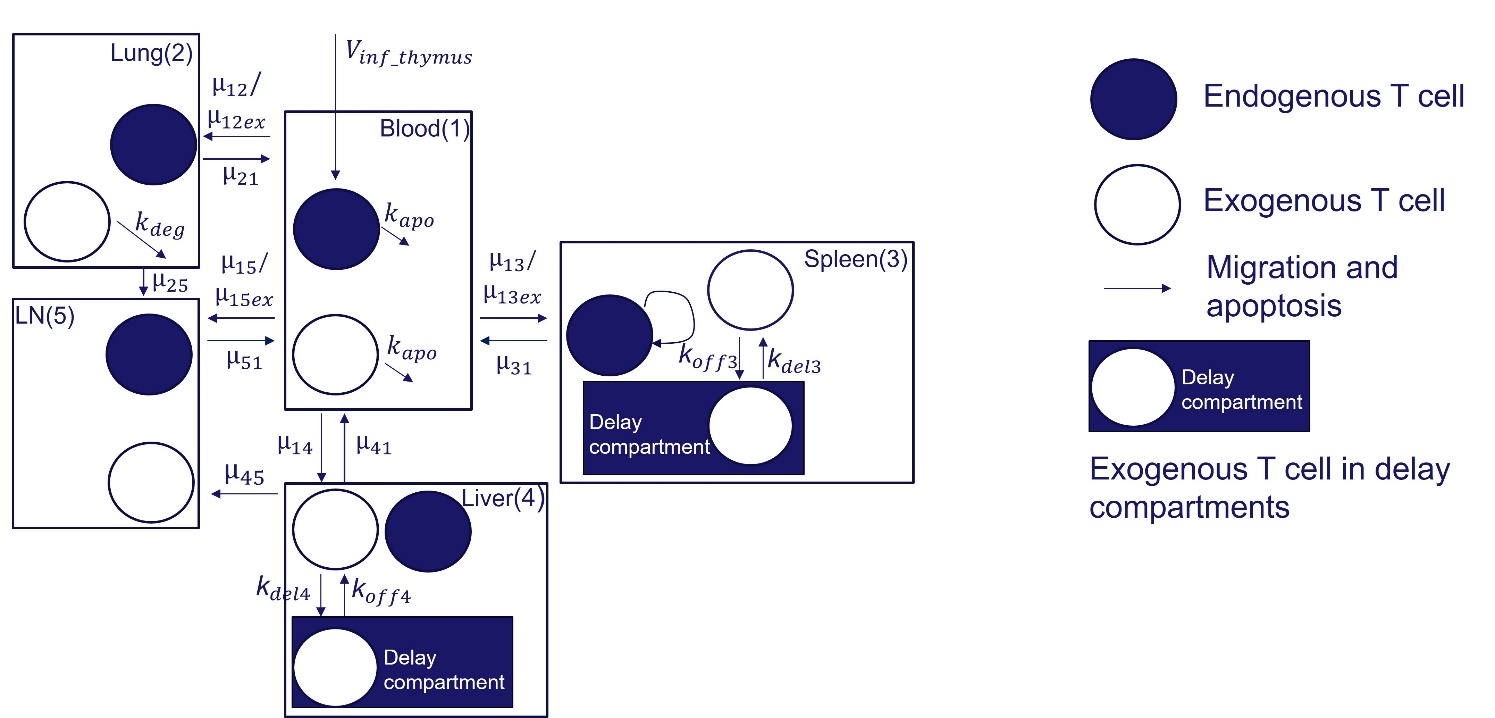


**Figure S2.** (A-E) Model calibration using data on exogenously administered T cells in: (A) blood, (B) lungs, (C) spleen, (D) lymph node, and (E) liver. Datapoints: Experimental data (mean ± error), calculated from (7). Curves: Model predictions; yellow shadows: 95% confidence intervals (CI). (F) Model calibration against data on endogenous T cells. Dark blue columns: model predictions; yellow columns: experimental data, calculated from (8), (12), (11) .


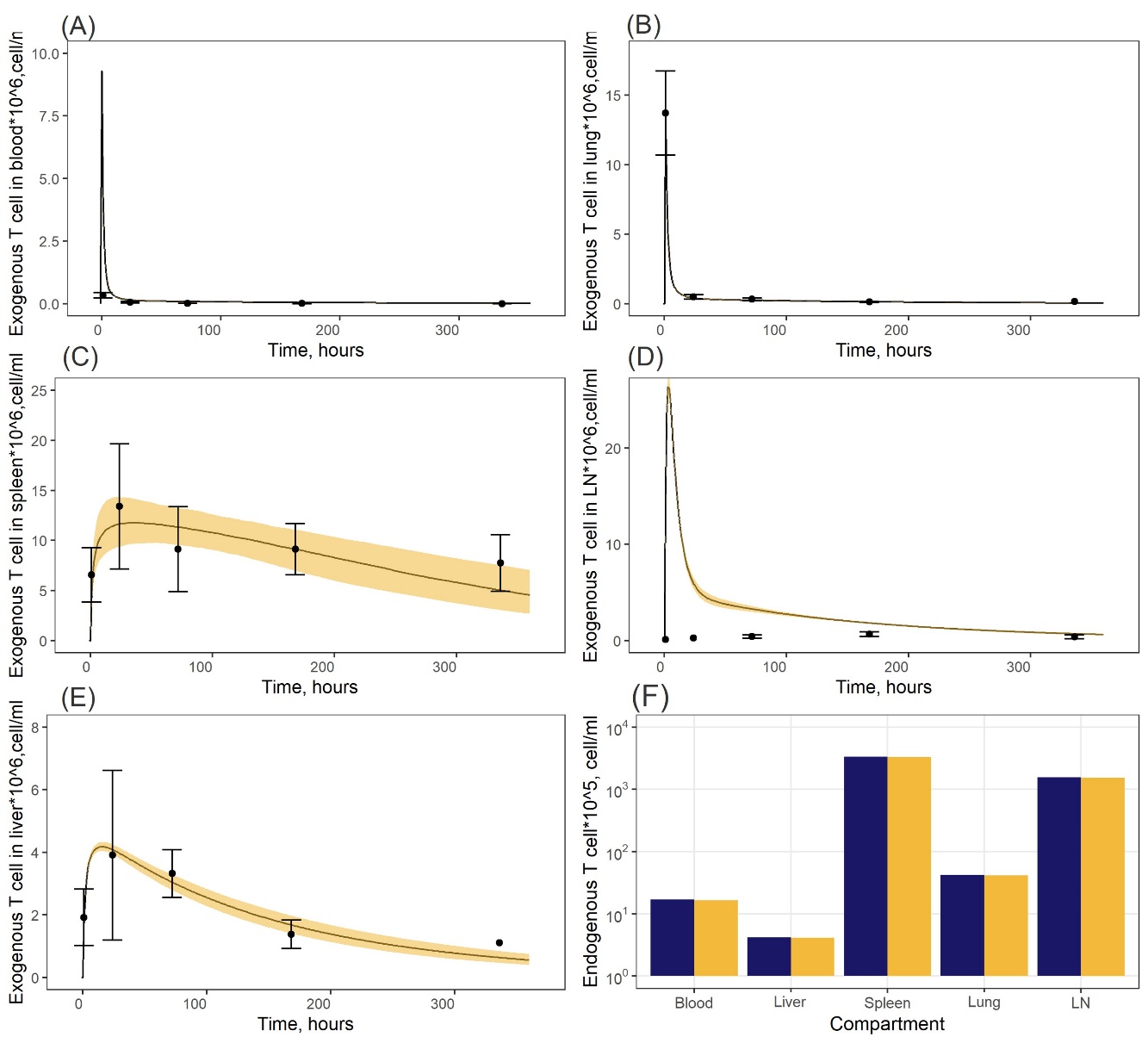


**Figure S3.** Global sensitivity analysis for levels of endogenous T cells in: (A) lungs, (B) liver, (C) generalized LN compartment, and (D) regional LN compartment, at steady-state.


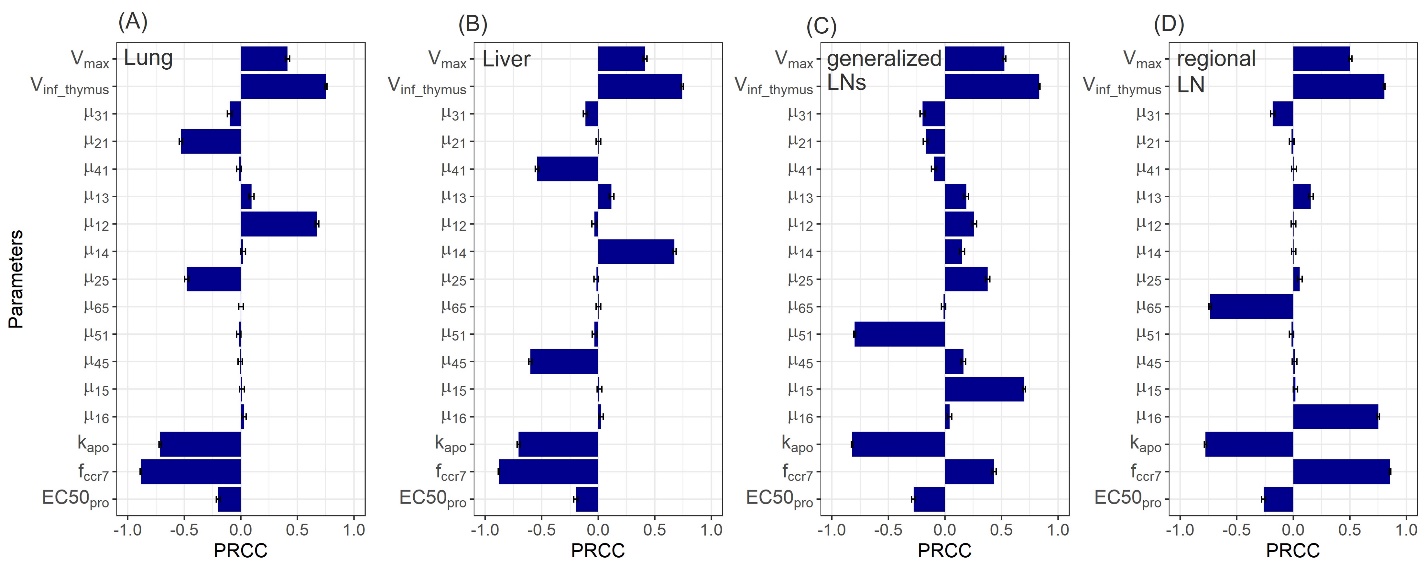


**Figure S4.** Local sensitivity analysis for AUC of exogenous T cells in: (A) lungs; and (B) generalized LN compartment.


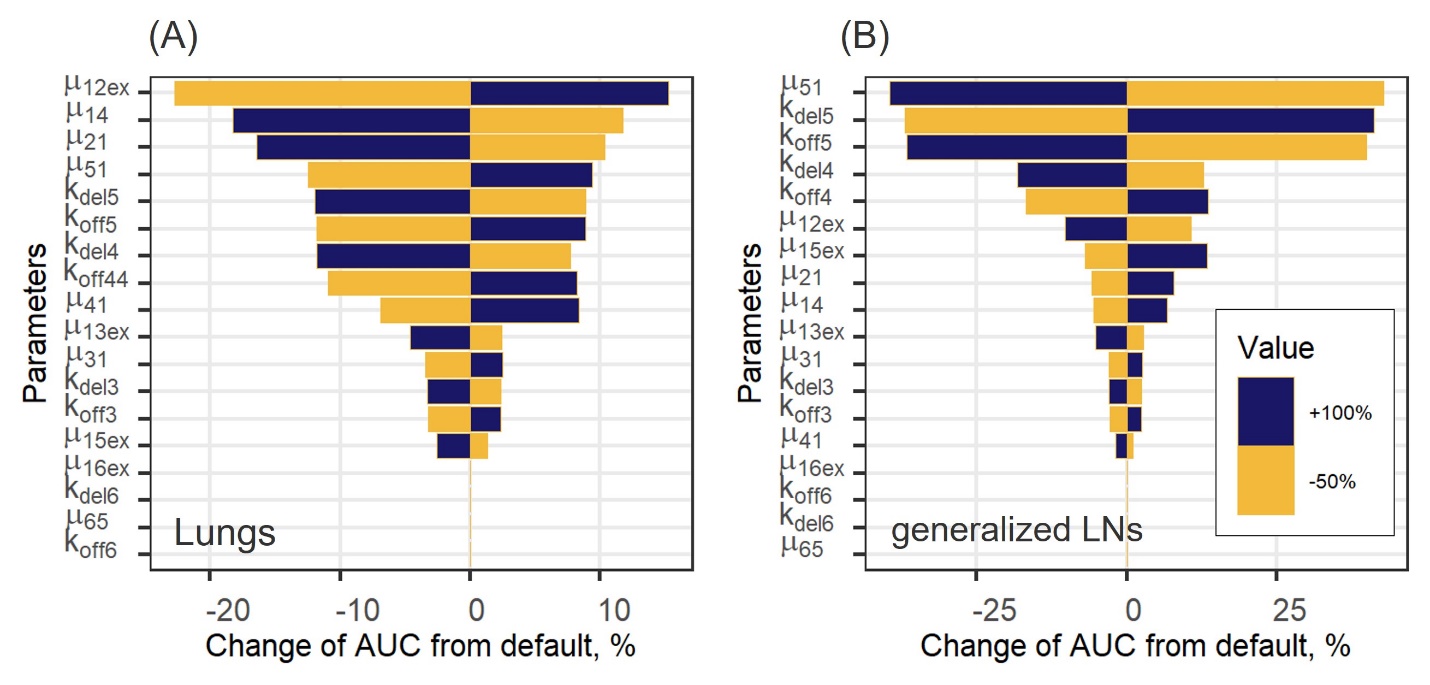


**Figure S5.** Local sensitivity analysis for *C_max_* of exogenous T cells in: (A) spleen; (B) liver; (C) regional LN compartment; (D) lungs; and (E) generalized LN compartment.


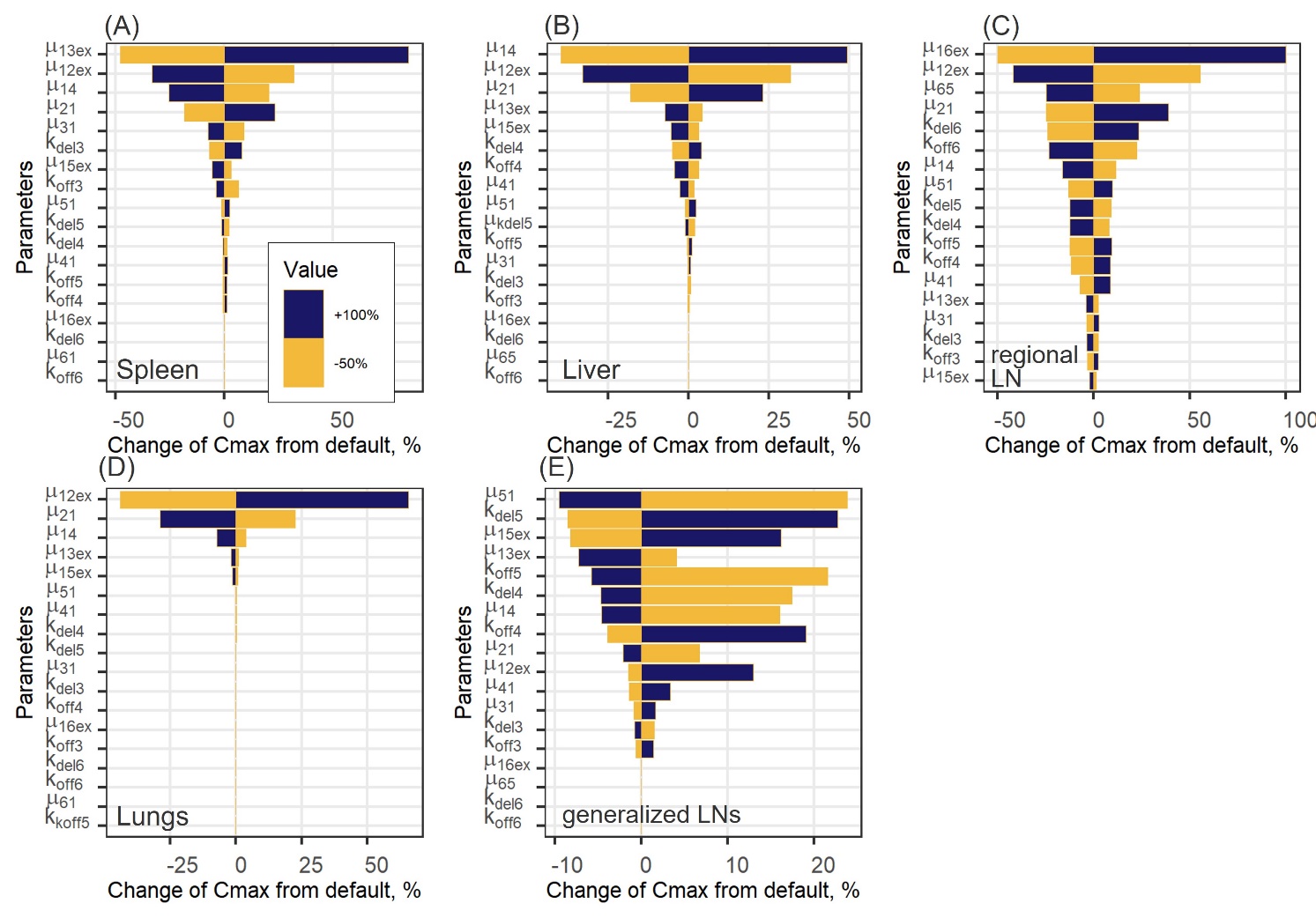


**Figure S6.** Kinetics of total exogenous T cell numbers. The total numbers of exogenously administered T cells were simulated after varying the following parameters: (A) rate constants related to delay compartments; (B) exogenous T cell inflow to organs. Dashed lines reflect simulations with default parameters.


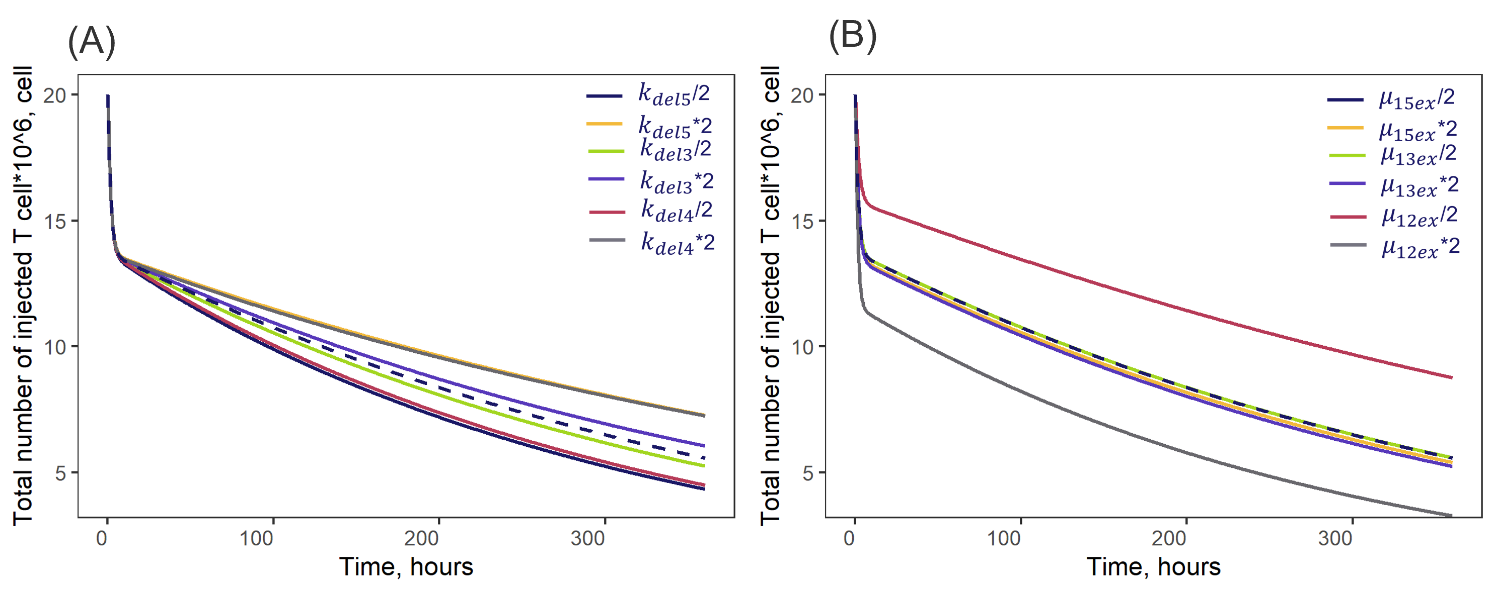


**Figure S7.** Kinetics of exogenously administered and endogenous T cell number in various compartments, for an infused exogenous T cell dose of 20.0*10^6^ cells. Dark blue lines: model simulation for exogenous T cell kinetics; dark red lines: endogenous T cell steady-state levels.


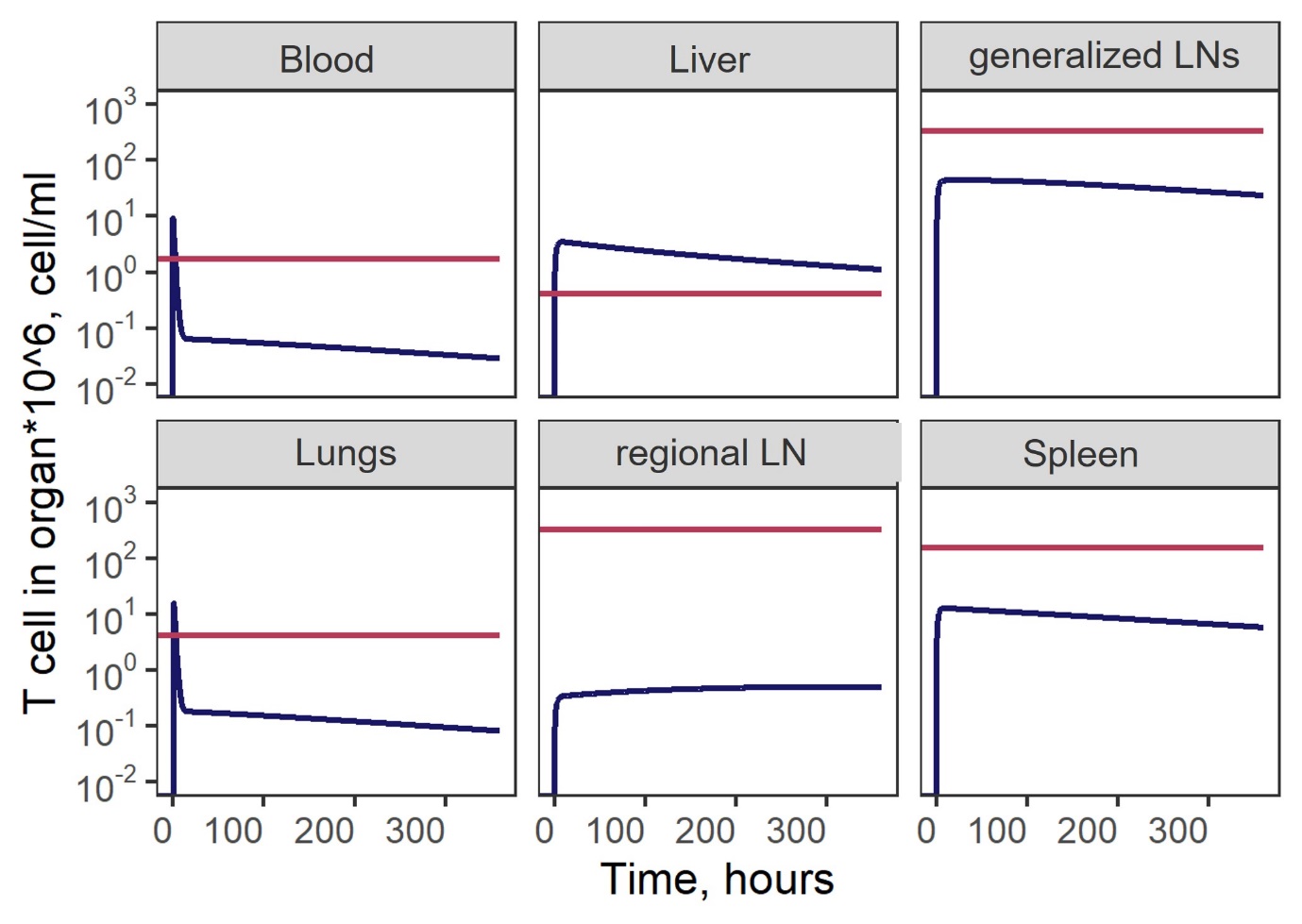


**References**

1. Dummer W, Niethammer AG, Baccala R, Lawson BR, Wagner N, Reisfeld RA, et al. T cell homeostatic proliferation elicits effective antitumor autoimmunity. J Clin Invest. 2002 Jul;110(2):185–92.

2. den Braber I, Mugwagwa T, Vrisekoop N, Westera L, Mögling R, de Boer AB, et al. Maintenance of peripheral naive T cells is sustained by thymus output in mice but not humans. Immunity. 2012 Feb 24;36(2):288–97.

3. Vianna PHO, Canto FB, Nogueira JS, Nunes CFCG, Bonomo AC, Fucs R. Critical influence of the thymus on peripheral T cell homeostasis. Immun Inflamm Dis. 2016 Dec;4(4):474–86.

4. Sta Maria NS, Khawli LA, Pachipulusu V, Lin SW, Zheng L, Cohrs D, et al. Spatio-temporal biodistribution of 89Zr-oxine labeled huLym-1-A-BB3z-CAR T-cells by PET imaging in a preclinical tumor model. Sci Rep. 2021 Jul 23;11(1):15077.

5. Maria NS, Khawli L, Lin S, Pachipulusu V, Zheng L, Cohrs D, et al. Simultaneous PET/MRI measurement of primary T cells biodistribution in naïve mice following adoptive cell transfer. Journal of Nuclear Medicine. 2019 May 1;60(supplement 1):277–277.

6. Leland P, Kumar D, Nimmagadda S, Bauer SR, Puri RK, Joshi BH. Characterization of chimeric antigen receptor modified T cells expressing scFv-IL-13Rα2 after radiolabeling with 89Zirconium oxine for PET imaging. J Transl Med. 2023 Jun 7;21:367.

7. Khot A, Matsueda S, Thomas VA, Koya RC, Shah DK. Measurement and Quantitative Characterization of Whole-Body Pharmacokinetics of Exogenously Administered T Cells in Mice. J Pharmacol Exp Ther. 2019 Mar;368(3):503–13.

8. Boyer SW, Rajendiran S, Beaudin AE, Smith-Berdan S, Muthuswamy PK, Perez-Cunningham J, et al. Clonal and Quantitative In Vivo Assessment of Hematopoietic Stem Cell Differentiation Reveals Strong Erythroid Potential of Multipotent Cells. Stem Cell Reports. 2019 Apr 9;12(4):801–15.

9. Wehrmann F, Lavelle JC, Collins CB, Tinega AN, Thurman JM, Burnham EL, et al. γδ T cells protect against LPS-induced lung injury. J Leukoc Biol. 2016 Feb;99(2):373–86.

10. Ma Q. Severe pneumonia induces immunosenescence of T cells in the lung of mice. Aging (Albany NY). 2023 Jul 24;15(14):7084-7097

11. Cosgrove J, Hustin LSP, de Boer RJ, Perié L. Hematopoiesis in numbers. Trends Immunol. 2021 Dec;42(12):1100–12.

12. Misumi I, Mitchell JE, Lund MM, Cullen JM, Lemon SM, Whitmire JK. T cells protect against hepatitis A virus infection and limit infection-induced liver injury. J Hepatol. 2021 Dec;75(6):1323–34.
